# Antibacterial Peptides in Dermatology–Strategies for Evaluation of Allergic Potential

**DOI:** 10.3390/molecules23020414

**Published:** 2018-02-14

**Authors:** Milena Deptuła, Anna Wardowska, Maria Dzierżyńska, Sylwia Rodziewicz-Motowidło, Michał Pikuła

**Affiliations:** 1 Department of Embryology, Medical University of Gdańsk, 80-211 Gdansk, Poland; milenadeptula@gumed.edu.pl; 2 Department of Clinical Immunology and Transplantology, Medical University of Gdańsk, 80-211 Gdansk, Poland; anna.wardowska@gumed.edu.pl; 3 Department of Biomedical Chemistry, Faculty of Chemistry, University of Gdańsk, 80-308 Gdansk, Poland; maria.smuzynska@ug.edu.pl (M.D.); s.rodziewicz-motowidlo@ug.edu.pl (S.R.-M.)

**Keywords:** peptides, antimicrobial peptides, immunogenicity, allergy, toxicity

## Abstract

During recent decades, the market for peptide-based drugs, including antimicrobial peptides, has vastly extended and evolved. These drugs can be useful in treatment of various types of disorders, e.g., cancer, autoimmune diseases, infections, and non-healing wounds. Although peptides are less immunogenic than other biologic therapeutics, they can still induce immune responses and cause allergies. It is important to evaluate the immunogenic and allergic potential of peptides before they are forwarded to the expensive stages of clinical trials. The process of the evaluation of immunogenicity and cytotoxicity is complicated, as in vitro models and bioinformatics tools cannot fully simulate situations in the clinic. Nevertheless, several potentially promising tests for the preclinical evaluation of peptide drugs have been implemented (e.g., cytotoxicity assays, the basophil activation test, and lymphocyte activation assays). In this review, we focus on strategies for evaluation of the allergic potential of peptide-based therapeutics.

## 1. Introduction

Peptides are small molecules that build from 50 or fewer amino acids [1]. They can be designed based on natural bioactive peptides/proteins present in plants, animals, and humans; naturally occurring fragments of enzymes; peptide hormones or host defense peptides; and even structures found in fungi, bacteria, or viruses [2,3,4,5]. Their advantages, e.g., good efficacy, safety, tolerability, high selectivity, potency, predictable metabolism, and standard synthetic protocols, make them promising candidates for use as drugs, and are thus of increasing interest in that field. Peptides generally have low oral bioavailability, they are susceptible to metabolizing factors, and they can be cleared from the circulation within minutes. Nowadays, alternatives to the oral route of administration, such as subcutaneous, intravenous, or intramuscular, have become accepted in clinical routines. Moreover, peptides that show high biological activity are taken into consideration as potential biopharmaceuticals [6,7,8]. The increasing interest in proteins and peptides as drug candidates may also be attributed to the development of chemical and biotechnological methods for large-scale manufacturing of proteins and polypeptides. In addition, currently available analytical methods make the roles of peptides in pathological conditions more comprehensible. Peptides are also easier to modify than standard organic compounds, and thus their propensity to be rapidly metabolized may be reduced. They may, for example, undergo N-terminal esterification (phosphoester) [9] or pegylation modifications [10], which make them resistant to exopeptidases. These modifications may also have an impact on the reduction of immunogenicity [11,12], and pegylation, which enlarges proteins, can reduce renal clearance [13].

Peptides are not only easy to modify but also to conjoin. Joining two active sequences may lead to a bifunctional compound, which is able to fight primary disease and help restore homeostasis (for example a conjoined antimicrobial peptide with a pro-proliferative sequence [14]). Moreover, the functional comprehensiveness and structural variety of peptides makes them excellent candidates for use in conjugations of biomolecules.

In the world of bioconjugate chemistry, antimicrobial peptides (AMP) hold a prominent position. As the increase in the emergence of multi-drug resistant pathogenic microorganisms has become one of the biggest problems in modern medicine, peptides seem to be a promising alternative to classic antimicrobial agents [15]. AMPs are a part of host defense mechanisms and mostly possess dual activity. Their functions are linked to their origin, i.e., the direct killing of microbes [16], the recruitment of leukocytes and the induction of cytokine/chemokine release [17], the promotion of angiogenesis [18], wound healing [19], mast cell degranulation [20], and the neutralization of endotoxins [21]. AMPs exert strong activity against various microbes (bacteria, fungi, and viruses), including multidrug-resistant strains. The risk of acquiring pathogen resistance to such bioactive compounds is low. However, peptides may be cytotoxic to human cells [22,23,24].

The story of therapeutic peptides begins with insulin [25] (manufactured by recombinant DNA technology in *E. coli* [26]) and oxytocin (produced by chemical synthesis elaborated by du Vigneaud in 1953 [27]), which are the most recognizable manufactured peptide hormones. Peptides show potency in treatment of cancer, asthma, neuropathic pain, stroke, diabetes, HIV, heart disease, and wound healing [14,28,29]. Some peptides also have immunoregulatory and anti-inflammatory properties, thanks to which they can be used in the treatment of autoimmune diseases [30,31]. Currently, approximately 140 peptides are undergoing various clinical trials, and more than 500 peptide compounds are being subjected to preclinical trials in order to become potential therapeutics. It is worth mentioning that the market for peptide and protein drugs makes up about 10% of the entire pharmaceutical market and, by 2015, the FDA had approved more than 60 peptide medicines [32,33]. In this mini-review, we would like to discuss peptide immunogenicity and the risk of inducing allergies, as well as methods potentially useful in the prediction of these features.

## 2. Adverse Reactions to Biological Drugs

Peptides, which are not classified as traditional biological drugs, can exert immunogenic properties similar to proteins [34]. Adverse reactions to biological products differ from those caused by standard chemotherapeutics. Unwanted effects are more common for chemically synthesized proteins and peptides than for compounds naturally present in the human body. A peptide’s influence on the immune system can be multi-directional and depends on host individual immune reactivity, dose, duration of treatment and dosing frequency, treatment scheme (other pharmaceuticals) and, last but not least, the type of the patient’s disease [35,36,37]. Product source can also have an impact on its immunogenicity: for example, recombinant human insulin is less immunogenic than porcine insulin [38]. The route of administration is important as well. Generally, the highest risk of inducing an immune response is after the subcutaneous route of administration, followed by intramuscular, intranasal, and intravenous routes [39,40]. Immunogenicity of peptides or proteins can potentially affect their efficiency and lead to adverse reactions such as allergy or hypersensivity [41]. Biologics may induce secretion of pro-inflammatory cytokines, as well as stimulate T cells, basophils/mast cells (allergic reactions), or neutrophils [36,42,43]. These compounds can also stimulate production of neutralizing or non-neutralizing anti-drug antibodies (ADAs). Neutralizing antibodies, present only in a small percentage of treated patients, cause negative consequences due to neutralization of a therapeutic product, thereby reducing its efficiency [39]. For instance, 40% of patients treated for multiple sclerosis (MS) with interferon beta (INF-β) develop ADAs (mostly neutralizing), which is associated with loss of efficiency of the INF-β treatment [44]. On the other hand, a majority of patients develop non-neutralizing antibodies, which do not significantly affect therapeutic effects of the drug [39]. Formation of ADAs can cause infusion reactions and anaphylaxis [45,46], immune complex-mediated diseases [47], and even such serious conditions as thrompocytopenia or pure red cell aplasia [48,49]. It is more likely that patients who develop ADAs will suffer from acute hypersensitivity reactions [50].

Although ADAs generally represent the IgG isotype, some complications may be caused by IgE-mediated immune responses. These responses may include local skin reactions or systemic reactions like anaphylaxis, which can potentially threaten a patient’s life. However, severe reactions are rare and more often associated with administration of xenopeptides or drug re-administration [50,51]. Moreover, some acute reactions can be antibody-independent and caused by cytokine release [50].

## 3. Tests Useful in Prediction of Peptide Immunogenicity and Allergic Potential

**Figure 1:**
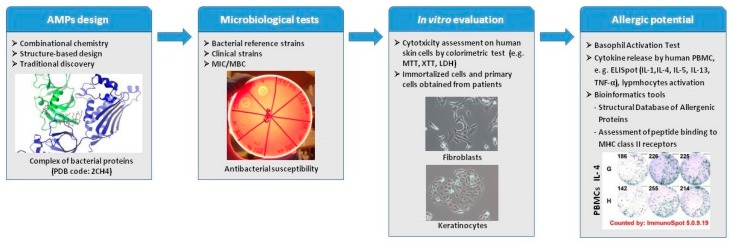
Preclinical evaluation of AMPs.

In general, peptides are less immunogenic than recombinant proteins and antibodies, but they can induce immune responses and allergies. The effects of peptides on the immune system depend on the compounds’ physicochemical properties and amino-acid sequence [52]. Although their features, like absorption and transport through biological membranes and barriers, are predictable, based on their physiochemical properties (such as water solubility, lipophilicity, proneness to forming H-bonds, chemical stability, and inclination to succumbing to proteolytic degradation [53]), their immunogenicity and risk of inducing allergy is difficult to predict. Based on our experience in evaluation of AMPs in wound healing, we can assume that there are a few in vitro test and bioinformatics tools that can be useful in evaluation of allergic potential and immunogenicity (Figure 1). Even though these tests cannot fully simulate immune responses in the clinic, they can be helpful in preclinical testing of different protein or peptide drugs [54,55]. In addition, it is important to use human cells, as the immune reactions observed in animal studies may differ significantly from human reactivity [42].

### 3.1. Peptide Cytotoxicity

In addition to the fact that peptides can be cytotoxic to human cells, there is a need to analyze their influence on living cells. Colorimetric methods, such as an MTT assay (and its modifications) or an LDH assay can be a first step in screening of peptide drug candidates. The MTT assay is based on living cells’ ability to reduce tetrazolium salts to formazan. The LDH test measures the activity of lactate dehydroganase (LDH), which is released by dead or membrane-damaged cells, in culture supernatants. This initial screening allows introduction of appropriate modifications to the tested peptides, leading to the strengthening of their biological activity or reduction of their side effects and toxicity [24]. Human skin cells can be used to evaluate the cytotoxicity of antimicrobial peptides. Barańska-Rybak et al. [56] showed that antimicrobial peptides can have different effects on human HaCaT keratinocytes, depending on their structure. In that study, most of the tested peptides were not toxic to human cells at their minimal inhibitory concentrations. Only lipopeptide exerted strong antiproliferative activity against HaCaT cells, which may be connected to its chemical properties including hydrophobicity. It is also crucial to choose an appropriate in vitro model for toxicity assessment. Transformed human skin cell lines, e.g., the HaCaT keratinocyte cell line, which reflect the properties of primary cells, provide reliable and reproducible results. They also prevent donor-to-donor variability from existing in primary cell lines isolated from human skin samples [14]. However, the significant variability between the reactivity of primary and transformed human cells lines cannot be excluded.

### 3.2. Basophil Activation Assay (BAT)

**Figure 2:**
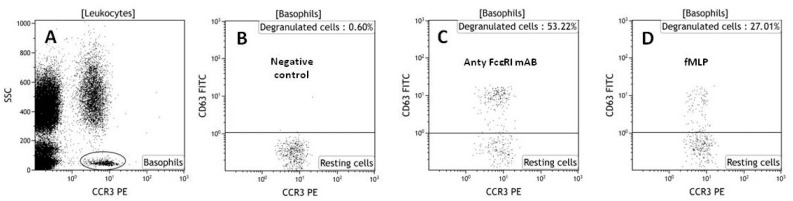
Representative dot plots (based on our own data) showing gating strategy of basophils based on low SSC-A values and high CCR-3 expression (**A**); Basophils were displayed in a graph with CCR3-PE on the x-axis and CD63 FITC on the y-axis; negative control (**B**); cells activated with stimulating antibodies (**C**); cells stimulated by bacterial tripeptide-fMLP (**D**).

The basophil activation test (Figure 2) allows the detection of hypersensitivity reactions in vitro by flow cytometry. It is a routine test used for the evaluation of the allergic potential of various drugs, including antibiotics [57]. It relies on flow cytometric identification and quantification of changes in activation of markers on the surface of basophils, detected with specific monoclonal antibodies coupled to fluorochromes [58]. The BAT is more expensive and technically more advanced than other available in vitro and in vivo tests, but it allows safe prediction of the allergenicity of different compounds [59]. It can be used to determine the allergic potential of new prospective drugs, like peptides and other biologics, and may be an alternative to provocation tests [58]. Flow cytometric analysis of activated basophils may be performed on whole blood or on basophils isolated by buffy coat centrifugation or dextran sedimentation. However, a whole blood assay is preferred, as it can be more efficient [60]. In the BAT protocol, whole blood basophils are challenged with the tested compound or one of the basophil activators anti-FcɛRI or fMLP, which activates basophils in an immunologic or nonimmunologic way. The expression of three markers is then checked: CCR3 (basophil population marker) and CD63 and CD203c (markers of basophil activation) [61]. In our laboratory, we have used the BAT test to evaluate the allergic potential of antimicrobial compounds (A20, camel, citropin). The results showed that antimicrobial peptides and peptidomimetics can have different allergic potentials. A20, also called Cystapep 1 (peptidomimetic), did not activate basophils obtained from healthy donors, but camel and citropin (peptides) had an allergic potential in hypersensivitive patients [54,61]. This shows that people suffering from allergies should be taken into consideration in preclinical testing of new potential peptide drugs.

### 3.3. Cytokine Assays and Lymphocyte Activation Analysis

Measurement of the release of Th2 response cytokines (IL-4, IL-5, IL-13) by human Peripheral Blood Mononuclear Cells (PBMCs), by the ELISPOT method, can also be used in preclinical testing. These cytokines are accountable for human allergic inflammation [62]. This evaluation method can be used in two ways. First, incubation of PBMCs with the tested peptide will show whether the compound can activate PBMCs and stimulate the release of Th2 cytokines, therefore indicating an allergic or immunogenic potential. Second, challenging of activated PBMCs (e.g., by Concanavalin A, a T-cell mitogen, which stimulates release of different cytokines) with the tested peptide can show whether it decreases secretion of cytokines and exerts immunomodulatory properties. Release of TNF-α, an early marker of immune system activation, by PBMCs can also be assessed [61]. The secretion of proinflammatory cytokines (i.e., TNF-α, IL-1 etc.) may indicate an inflammatory potential of the analyzed compound or even imply a higher risk of allergy induction. Another way to evaluate the immunogenic or allergic potential of the examined compounds is an assessment of activation marker expression on cells by flow cytometry analysis. This very useful and comprehensive method enables checking of whether selected PBMCs subpopulations activate in presence of the analyzed peptides. The majority of tests focus on various T cell subpopulations, as they play a pivotal role in immune responses; for example, T helper cells (Th), upon immunogenic peptide stimulation, increase expression of three major activation markers, namely CD25, CD69, and CD71 [63,64]. The cells participate in lymphocyte B activation and their antibodies participate in IgE production, thus contributing to an allergic reaction [65].

### 3.4. Bioinformatics Tools

As allergenic proteins from different sources can have similar sequences and structures, databases and bioinformatics search tools can be helpful in prediction of the allergic potential of peptides. The Structural Database of Allergenic Proteins (SDAP) is an online database that contains information on over 800 allergens and can be used to find structural and functional similarities of designed peptides to known allergens [66]. The property distance index (PD), calculated based on comparison of a peptide sequence and sequences in SDAP, allows prediction of IgE cross-reactivity and allergic potential. According to literature data, a PD value cutoff between 7.5 and 9.0 is suitable for determination of peptides with similar properties. Peptides with no similarities to SDAP have PD values much higher than 10 [54,67]. Our previous study showed that synthetically produced antimicrobial peptides (citropin and camel) are structurally similar to common environmental allergens. Combining the results of the database search with a BAT test proved to be useful in the assessment of the risk of allergic reaction induction by potential peptide drugs [50].

Another strategy for allergic potential evaluation may be an assessment of peptide binding to MHC class II receptors. HLA class II molecules mediate the response of CD4 T-cells to exogenously administrated proteins. [68]. Dhanda et al. [69] evaluated a new computational tool for prediction of “de-immunized” peptides. Their approach was based on the fact that HLA binding is crucial for T-cell immunogenicity and, if HLA binding is decreased or abolished, there is an expected decrease in protein/peptide immunogenicity. Their method predicts binding regions on a protein sequence and identifies residue substitutions that can reduce HLA binding. They used this program to select factor VII analogues with reduced immunogenicity. This algorithm correctly predicted two immunogenic peptides for which in vitro tests on human PBMCs showed positive responses in 46% of cultures. In the next step, they evaluated the immunogenicity of seven substitutions that were predicted to simultaneously reduce HLA binding for both peptides. In in vitro tests they showed immunogenicity in 21.4% of PBMCs cultures. Their results show that this online tool can be useful in searching for peptides with reduced immunogenicity.

## 4. Conclusions

The immunogenicity of peptide-based and protein drugs, which can affect their efficiency and cause unwanted side effects, is an important consideration in the biological evaluation of these potential therapeutics. It is crucial to check their immunogenic or allergic potential before these drug candidates are subjected to expensive clinical trials. In vitro immunological tests, as well as bioinformatics tools and databases, despite being a simplification of naturally occurring systemic reactions, comprise a valuable tool in the assessment of potential peptide therapeutics. It is therefore of great value to elaborate and implement new, more accurate, preclinical tests based on, e.g., 3D tissue models, organ-on-a-chip, or lymphoid tissue-like organoids.
